# A Canadian Perspective on the Challenges for Delivery of Curative-Intent Therapy in Stage III Unresectable Non-Small Cell Lung Cancer

**DOI:** 10.3390/curroncol28030151

**Published:** 2021-04-24

**Authors:** Anthony Brade, Kevin Jao, Simon Yu, Parneet Cheema, Sarah Doucette, Anna Christofides, Devin Schellenberg

**Affiliations:** 1 Department of Radiation Oncology, Peel Regional Cancer Centre, Mississauga, ON L5M 2N1, Canada; 2 Department of Hematology and Oncology, Hôpital du Sacré-Coeur de Montréal, Montreal, QC H4J 1C5, Canada; kevin.jao@mail.mcgill.ca; 3 Department of Medicine, Burnaby Hospital Cancer Centre, Burnaby, BC V5G 2X6, Canada; Simon.yu@fraserhealth.ca; 4 Department of Medicine, Division of Medical Oncology, University of Toronto, Toronto, ON M5S 3H2, Canada; Parneet.cheema@williamoslerhs.ca; 5 William Osler Health System, Brampton, ON L6R 3J7, Canada; 6 Senior Medical Writer, IMPACT Medicom Inc., Toronto, ON M6S 3K2, Canada; sarah@impactmedicom.com (S.D.); anna@impactmedicom.com (A.C.); 7 Department of Radiation Oncology, BC Cancer Agency, Surrey, BC V2V 1Z2, Canada; dschellenberg@bccancer.bc.ca

**Keywords:** stage III non-small cell lung cancer, inoperable, curative-intent, chemoradiation therapy, immunotherapy, care pathway

## Abstract

Stage III non-small cell lung cancer (NSCLC) comprises a highly heterogenous group of patients with regards to patient fitness and tumour size and distribution, resulting in a wide range of treatment goals and therapy options. Curative-intent multimodality treatment should be considered in all patients with stage III NSCLC. For patients with unresectable disease who are fit, have adequate lung function, and have a disease that can be encompassed within a radical radiation volume, concurrent chemoradiation therapy (cCRT) is the standard of care and can produce cure rates of 20–30%. Recently, consolidation immunotherapy with durvalumab has been recognized as the standard of care following cCRT based on significant improvement rates in overall survival at 4 years. The large heterogeneity of the stage III NSCLC population, along with the need for extensive staging procedures, multidisciplinary care, intensive cCRT, and now consolidation therapy makes the delivery of timely and optimal treatment for these patients complex. Several logistical, communication, and education factors hinder the delivery of guideline-recommended care to patients with stage III unresectable NSCLC. This commentary discusses the potential challenges patients may encounter at different points along their care pathway that can interfere with delivery of curative-intent therapy and suggests strategies for improving care delivery.

## 1. Introduction

Lung cancer is the most frequently diagnosed cancer in Canada, with 29,800 cases projected in 2020, and it is also the leading cause of cancer death [1]. Non-small cell lung cancer (NSCLC) accounts for approximately 85% of newly diagnosed cases, with the majority of these patients presenting with advanced disease; almost half of patients have stage IV and around one fifth have stage III at diagnosis [2].

Patients with stage IV NSCLC are not curable and typically have poor prognosis, with a historical 5-year survival rate of 10% [3]. Palliation of symptoms, quality of life improvements, and prolonging survival are thus the main treatment goals for these patients [4]. For stage III NSCLC, 5-year survival rates range between 13 and 36%, depending on substage of disease [3]. The optimal treatment plan for these patients is complex owing to the heterogeneity in performance status and tumour size, location(s), and resectability between patients. Unlike patients with stage IV NSCLC, patients with stage III disease should be evaluated for curative-intent therapy.

Curative-intent treatment for stage III NSCLC is multimodal, consisting of a combination of chemotherapy, radiation, and/or surgical resection, although the optimal sequence and modality is debated and highly case-specific [5]. The extensive staging work-up required to assess the feasibility for curative-intent treatment, and the need for consultation with a multidisciplinary team, further complicates the optimal, individualized management of stage III patients. In patients with unresectable disease who are fit (Eastern Cooperative Oncology Group Performance Status 0–1), have adequate lung function, and have a disease that can be encompassed within a radical radiation volume, chemoradiation therapy (CRT) using platinum-based chemotherapy is the standard of care [6]. Concurrent CRT (cCRT) is typically favoured for these patients, owing to multiple clinical studies showing its superiority to sequential CRT in stage III patients [7].

As cCRT can be curative in 20–30% of patients with stage III NSCLC, it is critical that newly diagnosed patients be assessed for cCRT treatment eligibility [8]. However, even with cCRT, there is considerable room to improve survival outcomes. Within the last few years, immunotherapy has been introduced into stage III treatment regimens as a consolidation therapy following cCRT and has shown the potential to substantially improve survival outcomes. In the 4-year update of the phase III PACIFIC trial, durvalumab following cCRT led to a significant improvement in overall survival (OS) versus placebo in patients with stage III NSCLC (median OS: 47.5 vs. 29.1 months; HR: 0.71 (95% CI, 0.57 to 0.88)) [9]. Progression-free survival (PFS) was also significantly improved (median PFS: 17.2 vs. 5.6 months; HR: 0.55 (95% CI, 0.44 to 0.67); *p* < 0.001)), with only a 4% increase in grade 3/4 adverse events from the addition of durvalumab [9,10]. This marks a major milestone for the treatment of stage III NSCLC, and as such, consolidation durvalumab is recommended as a standard of care in international guidelines for patients with stage III locally advanced, unresectable NSCLC whose disease has not progressed following platinum-based cCRT [11,12,13]. As a result, durvalumab has since been approved by Health Canada (May 2018) and is currently reimbursed in all provinces for this indication [14].

Despite the practice-changing results of the PACIFIC study and availability of durvalumab for patients with stage III unresectable NSCLC, the percentage of patients receiving curative-intent cCRT is small. Real-world data from the UK suggest that only 20–30% of stage III patients receive curative-intent CRT [5]. Similar low rates have been reported in Canada, with evidence of significant variation in the proportion of stage III patients who receive cCRT between provincial jurisdictions [15,16,17]. Multiple factors may contribute to the small proportion of stage III NSCLC patients receiving cCRT in clinical practice, including eligibility based on patient fitness. However, a recent retrospective chart review from British Columbia, in the year following durvalumab approval, indicated that even in patients who were medically eligible and able to receive durvalumab, 36% of patients did not receive this consolidation therapy following cCRT [18]. This suggests that other factors may hinder the ability for physicians to deliver guideline-recommended cCRT followed by durvalumab in patients with stage III NSCLC. This may include the logistical and communication challenges that arise from the multidisciplinary management of stage III patients receiving cCRT, and the additional complexities introduced by consolidation durvalumab to the care path.

To improve outcomes for patients with stage III unresectable NSCLC, it is therefore important to evaluate the multidisciplinary care pathway for these patients, which includes consultation with thoracic surgeons, radiation oncologists, and medical oncologists, and consider how navigation through this pathway can be improved to allow optimal treatment delivery. This commentary presents the authors’ views on what potential challenges patients with stage III unresectable NSCLC may encounter at different points in their cancer journey, that can interfere with delivery of curative-intent therapy. Discussion is focused on challenges related to diagnosis and staging, treatment planning, and initiation and management of cCRT and immunotherapy, with suggested strategies to overcome these challenges.

## 2. Challenges in Delivery of Curative-Intent Chemoradiation Therapy

### 2.1. Diagnosis and Staging

Extensive delay in treatment initiation has been correlated with increased mortality for several cancers [19], including lung cancer [20,21], with studies indicating late diagnosis as a major contributor to this delay [22,23]. Poor outcomes related to delay in definitive diagnosis may be caused by the patient experiencing disease progression or a decline in function while awaiting staging tests, and as a result, a patient who was initially eligible for curative-intent therapy may become ineligible. As a delay in diagnosis can also impact patient well-being by increasing anxiety [24], a focus on reducing diagnosis time is needed for optimal patient-centered care.

The process of accurately diagnosing stage III NSCLC can be time-intensive but is critical for identifying those patients who may be eligible for curative-intent therapy. Generally, the pathway to a definitive diagnosis begins with the patient presenting with symptoms to a health care provider (HCP), such as a general practitioner, respirologist, or emergency department physician. The initial HCP would order an imaging test after which, if primary bronchogenic carcinoma is suspected, would require extensive work-up to confirm diagnosis, staging, and provide information for treatment planning. The pathway after an abnormal imaging finding may differ by region, but typically involves referral to a respirologist or thoracic surgeon. Ideally, these specialists would be responsible for ordering additional tests before referral to an oncologist, although this is not always the case in practice. Further testing may include a computed tomography (CT) scan if not previously done, positron emission tomography (PET), bronchoscopy, cytology from bronchoalveolar lavage or pathology assessment of tissue biopsy, brain imaging (magnetic resonance imaging or CT), invasive mediastinal staging (endobronchial ultrasound (EBUS) or mediastinoscopy), and pulmonary function tests.

Some lung cancer guidelines, such as those from Nordic countries, suggest diagnostic work-up be completed within 26–30 days of referral, with an additional 7–15 days before treatment initiation [25]. Cancer Care Ontario has also proposed that a similar target of 28 days from initial referral to diagnosis be met in 65% of newly diagnosed lung cancer patients [26]. However, past studies evaluating the timing of diagnosis and treatment in multiple jurisdictions, including Canada, indicate that these timelines are difficult to achieve [27]. A cross-jurisdiction study through the International Cancer Benchmarking Partnership evaluated lung cancer patients enrolled in local registries between 2012 and 2015, in countries with similar healthcare access (UK, Nordic countries, and Canada) [27]. In this study, the median time from first presentation to healthcare to diagnosis date ranged from 28 to 87 days, with the Canadian provinces evaluated (Manitoba and Ontario) being among the jurisdictions with the longest reported time intervals (87 and 57 days, respectively).

Access to imaging tests is a common barrier to achieving timely diagnosis and staging of lung cancer [28]. For example, PET is not available at all centres in Canada, with some patients living in remote regions having to travel large distances to get PET CT. In addition, without triaging protocols in place to expedite access to imaging for patients with suspected lung cancer, patients may experience long wait times for an imaging appointment. Other factors contributing to delay in diagnosis at some institutions include insufficient availability of physician specialists, such as radiologists, pathologists, and experts in performing EBUS, as well as a shortage of other human resources, such as administration clerks [29,30]. Communication challenges can also contribute to diagnosis delays [26], which may be particularly true when patients require tests performed at multiple locations. Inadequate education of nearby referring physicians on local care pathways and work-up requirements can also contribute to delays in diagnosis [28]. For instance, lack of education or readily accessible guidelines may result in early referral to medical or radiation oncologists with incomplete work-up, making initial consultations with these specialists inefficient. Providing referring physicians with the tools they need to refer patients to the right specialists is critical for optimal and timely delivery of care.

Although institutions may share some challenges in achieving timely diagnosis and staging of lung cancer, internal quality assurance studies must be performed at individual centres to ensure their unique challenges are addressed. Several centres in Canada have performed quality assurance investigations of their lung cancer care pathway from onset of symptoms to initial treatment and have published articles detailing strategies that were effective in improving diagnosis time [26,28,31,32,33]. The most common strategy applied by all centres was the use of centralized referral centres/diagnostic assessment programs and a nurse navigator to help triage patients to the appropriate pathway and coordinate the many investigations needed for diagnosis and staging. Referral programs focused on streamlining diagnostic imaging by scheduling multiple tests on the same day where possible [26,32], improving access to test result reporting [26], pre-ordering or holding designated slots for tests [28,32,33], and triaging testing based on urgency [28,33]. Other strategies focused on education, including increasing awareness of the diagnostic assessment program to the referral base [28], and informing patients of their care path, allowing them to take ownership of their journey [26,32]. Implementation of integrated electronic information systems for performance monitoring and patient tracking were also used [26]. Together, these strategies may help address some of the challenges in testing access, timely workup, and communication to patients and between specialties.

Thus far, this section has focused on improving the care pathway for diagnosis and staging of lung cancer in a symptomatic patient. However, it is also important to consider screening programs in asymptomatic individuals with a high-risk of developing lung cancer as a strategy for improving timely diagnosis at earlier stages of disease when curative-intent therapy is more feasible. Two large randomized controlled trials have reported a substantial reduction in lung cancer mortality, as well as a lower presentation of incurable stage IV disease at diagnosis, for individuals receiving low-dose CT screening tests compared to those who did not [34,35]. Based on this data, the Canadian Task Force on Preventative Health Care recommends annual low-dose CT screening, up to three consecutive times, in adults aged 55–74 who currently smoke or have quit smoking less than 15 years ago, and with at least a 30 pack-year smoking history [36]. Despite these guidelines, lung cancer screening programs are lacking in all Canadian provinces, and efforts to implement such a program can play a role in improving the care pathway for all lung cancer patients.

At the time of writing, with the world having just marked the one-year anniversary of the COVID-19 pandemic, we cannot ignore the additional challenges that the SARS-CoV-2 virus has presented in achieving timely lung cancer diagnosis and treatment. Many factors have contributed to delays in lung cancer diagnosis during this time, including decreased access to diagnostic testing and specialist consultation caused by reallocation of resources to manage the surge of COVID-19 cases, precautions in performing diagnostic tests that generate aerosols, overlap between symptoms of COVID-19 and lung cancer, and a general reluctance of patients to interact with healthcare facilities [37]. As the pandemic continues, and at the point it is over, we are likely to face a surge in lung cancer investigations and cases, further emphasizing the need for centres to assess and optimize care pathways to recover some of the damages caused by COVID-19.

### 2.2. Treatment Planning

Ideally, after work-up is complete and diagnosis of stage III NSCLC is confirmed, patients potentially eligible for curative-intent treatment should be seen by both radiation and medical oncologists, as well as a thoracic surgeon if not already seen, to discuss treatment planning. This should occur within a short timeframe, as a long time interval from diagnosis to consultation can not only cause PET or CT scans to be outdated, leading to difficulty in treatment planning, but can allow the tumour to progress such that it is too large or widespread for radical treatment [21]. There are many factors that may play a role in delayed consultation with oncologists, with the degree at which each factor contributes to this delay likely varying significantly by geographical location.

Like the diagnosis and staging phase of care, a focus on strong education and clear communication between referring physicians, specialists, administrative personnel, and patients is necessary to ensure efficient referral pathways. The order of referral may depend on regional practices, appointment availability, and whether certain criteria may exclude the patient from curative-intent treatment. For example, pulmonary function test results may exclude eligibility for radical radiotherapy, and poor performance status or specific comorbidities may exclude eligibility for chemotherapy, both affecting how patients are triaged. Generally, referral to a radiation oncologist first can be beneficial as they are able to determine whether the patient’s tumour can be encompassed within a radical radiation volume, which is a deciding factor for eligibility to curative-intent therapy.

Treatment planning for stage III NSCLC can be complex for several reasons. Firstly, the distinction between what is considered resectable can be unclear. Technically speaking, any patient with stage IIIA NSCLC and pathologically confirmed N2 involvement may be potentially resectable [38]; however, there is a wide variation in the volume and extensiveness of nodal involvement which may impact choice of multimodal therapy [39]. A general consensus of what may be considered resectable in stage IIIA disease is discrete, easily measurable, and defined nodal involvement, that is free from major mediastinal structures, and where no individual lymph node measures > 3 cm [5]. Approximately 15% of stage III NSCLC cases are potentially eligible for surgical resection [15]. However, even in those patients, the optimal multimodality treatment remains unclear, as studies comparing multiple surgery-based regimens with CRT have not shown significant differences in OS [5,38,40,41]. The introduction of consolidation immunotherapy following cCRT adds another layer of complexity to the question of optimal treatment for stage III resectable NSCLC. Given that immunotherapy consolidation after cCRT has shown the potential to significantly improve survival outcomes (vs. placebo) [42], studies comparing this treatment regimen to cCRT plus surgery are needed. For patients with stage III NSCLC who are deemed unresectable, determination of whether a patient is eligible for curative-intent cCRT is also complex, as there are several factors that may influence a patient’s fitness for cCRT, and comprehensive guidelines to assess fitness do not exist [43]. This is illustrated by the wide variation of cCRT use within Canadian jurisdictions, suggesting there is likely substantial variation in willingness to proceed with cCRT [15,17].

Due to the significant complexity in treating patients with stage III NSCLC, multidisciplinary collaboration is often required to carefully weigh the benefits and risks of each regimen and decide on the optimal course of treatment for each patient. Several international guidelines recommend the use of a multidisciplinary team (MDT) for lung cancer management, composed of thoracic surgeons, radiation oncologists, medical oncologists, and other specialists and allied health professionals [11,44]. Lung cancer management through MDTs has been associated with improved outcomes and survival, and a greater likelihood of delivering guideline-recommended therapy [45].

This emphasizes the need for MDTs in the lung cancer care pathway, and that lack of access to an MDT or poor communication within the MDT could challenge delivery of optimal care. MDT collaboration can take several forms. They can be more formal interactions, consisting of regularly scheduled meetings, case conferences, or tumour boards, conducted virtually or in person. However, MDT meetings may not be accessible at some centres, and when available, they may only be able to cover more controversial cases. This may be particularly true in community-based settings where co-location, financial disincentives, and time constraints have been cited as major deterrents for MDT care [46]. When formal MDT interactions are not available, it is imperative that strong lines of communication between multiple specialties be established, whether they work in the same facility where in-person discussion is feasible, or whether members agree to communicate via email, phone, or virtual platform. Prior to the COVID-19 pandemic, issues of comfort with and quality of virtual platforms may have led to a reluctance to participate in remote tumour boards. However, the pandemic has forced centres to upgrade infrastructures to support virtual collaboration, and the reliance on virtual tools has increased physicians’ experience with them. This has increased the opportunity for MDT participation remotely by reducing the travel, time, and financial strain associated with MDT attendance.

### 2.3. Chemoradiation: Treatment Initiation and Management

Once a decision to treat a patient with curative-intent cCRT is made, therapy initiation must be scheduled and coordinated between radiation and medical oncologists. The most favourable scenario for patients would be to receive both chemotherapy and radiation therapy at the same local facility. However, this may be a challenge for patients living in rural or remote areas where community hospitals may not have access to radiation therapy. Indeed, in some jurisdictions, increased geographical distance from treatment centres, as well as lower socioeconomic status, can challenge the ability to deliver cCRT [47,48]. It is therefore important for oncologists and/or allied health professionals to have a clear understanding of available patient support programs that can offer overnight lodging and transportation for patients who require it. In cases where patients are receiving treatment at two different centres, seamless communication and coordination between the centres is essential for delivery of efficient care and adapting to unanticipated events.

Interruptions in planned radiation therapy can also challenge optimal delivery of care, as there is evidence to support that OS in patients with advanced-stage disease worsens with each cumulative interval of delay [49]. Disruptions in scheduled radiotherapy may occur due to machine and staff availability, public holidays, transportation challenges, concurrent illness or management of adverse events during treatment, and personal patient circumstances [50]. The Royal College of Radiologists provides strategies for prevention and management of treatment interruptions to optimize clinical outcomes for patients. Their published guidelines include recommendations for planning based on the most common reasons for treatment delay, as well as suggestions to compensate for treatment delays that aim for the completion of the radiotherapy course to be as close as possible to the initially planned date [50].

Advances in radiation technology, such as the introduction of intensity-modulated radiation therapy (IMRT), have not only expanded the types of patients that can be treated with cCRT, allowing more patients with stage IIIB and/or larger tumours to undergo curative-intent therapy, but have also led to a reduction in severe toxicities such as pneumonitis [51]. However, it remains important to closely monitor and manage adverse events in these patients to improve quality of life and increase the likelihood that patients are fit enough to receive survival-extending consolidation immunotherapy. Specific recommendations for management of acute and late toxicities of cCRT are reviewed elsewhere [52].

### 2.4. Immunotherapy: Treatment Initiation and Management

The PACIFIC trial has demonstrated that consolidation therapy with durvalumab every two weeks for up to 12 months following cCRT improves PFS and OS for patients with stage III NSCLC [42]. However, inadequate patient education on the potential benefits of durvalumab can act as a barrier to its uptake post-cCRT. Discussing the benefits of consolidation durvalumab with patients during initial cCRT planning allows patients to prepare for treatment mentally and physically, which may reduce the number of patients declining, delaying, or missing consolidation therapy once cCRT is complete. This may be especially important for patients who have difficulty travelling to centres for durvalumab infusion.

One factor that may benefit compliance to consolidation durvalumab is adjusting the dosing schedule of durvalumab to a fixed dose every four weeks, rather than a weight-based dose every two weeks as is currently indicated in the product monograph for patients with stage III NSCLC [53]. Administration of a 1500 mg fixed dose every four weeks in patients weighing more than 30 kg is consistent with the approved dosing for durvalumab in extensive-stage small cell lung cancer [53]. This dosage and schedule is based on that used in the CASPIAN trial which showed improved survival for the addition of durvalumab to platinum-etoposide compared to platinum-etoposide alone in extensive-stage small cell lung cancer [54]. In this trial, safety results were consistent with the known safety profiles of all drugs received [54]. As of November 2020, this fixed dosing schedule has been approved by the U.S. Food and Drug Administration for all durvalumab indications [55]. As the pharmacology for many immune checkpoint inhibitors can allow for less frequent dosing than what is currently used, guidelines for the treatment of lung cancer during the COVID-19 pandemic also suggest durvalumab dosing can be extended [56]. Indeed, this dosing schedule has been adopted in many centres across Canada during the COVID-19 pandemic to reduce travel times to and from the hospital and to reduce potential COVID-19 exposure. Real-world data evaluating patients who received a fixed dose of durvalumab every four weeks during the COVID-19 pandemic will be beneficial to confirm the efficacy and safety of fixed dosing in this population.

As the indication for durvalumab is specific to patients with stage III NSCLC with non-progressive disease post-cCRT, some form of radiological assessment post-cCRT is required to determine eligibility for durvalumab consolidation. Long wait times for CT scans can thus pose a challenge in accessing durvalumab post-cCRT. Early planning to book a CT scan at the time cCRT is complete can ensure patients can begin consolidation durvalumab within the 42-day timeframe used in the PACIFIC study. Considerations could also be made for alternatives to CT, such as chest x-rays, or use of the radiation oncologist’s notations from the last cone beam findings. A promptly organized CT scan is especially important in some Canadian jurisdictions that require durvalumab to be started within 42 days of cCRT for reimbursement eligibility and is also critical for patients who receive their radiation in another city.

Timely imaging and durvalumab initiation post-cCRT may be particularly important as the PACIFIC trial showed a signal for improved outcomes in patients who received durvalumab consolidation within 2 weeks of completing cCRT, compared with patients who initiated durvalumab after this time point [42]. However, it is unclear whether this improvement is a result of earlier initiation of durvalumab or whether it is due to these patients having a better overall health status. In addition, real-world data showing the experience with durvalumab consolidation in Quebec did not find that a delay in durvalumab initiation beyond 42 days had an impact on survival [57].

At the point of durvalumab initiation, patients may continue care with the medical oncologist who oversaw their chemotherapy or care may be transferred to a local medical oncologist or general practitioner in oncology (GPO). Poor communication in coordinating this transition could result in delayed initiation of durvalumab. In these cases, a nurse or clerical navigator could be beneficial for ensuring continuity in care. It is also important for the physicians involved in the care of patients to have an upfront discussion regarding responsibilities for follow-up. In some regions, follow-up responsibilities may fall entirely on the medical oncologist or GPO. This has implications for workload and adverse event management. The Saskatchewan Cancer Agency has guidelines for follow-up in patients treated with cCRT which recommend patients be followed by both the medical and radiation oncologists while on consolidation immunotherapy for the first year following completion of cCRT [58]. Subsequently, the radiation oncologist can discharge the patient and the medical oncologist can follow them for an additional 2 years after immunotherapy completion.

Multi-specialty follow-up is especially important for the diagnosis and management of pneumonitis, which can be life-threatening. In the PACIFIC trial, any grade pneumonitis occurred at a rate of 33.9% in patients receiving durvalumab, with grade ≥ 3 pneumonitis occurring in 3.4% (24.8 and 2.6% in the placebo arm, respectively) [10]. Real-world data have reported frequencies of any grade and grade ≥ 3 pneumonitis of 20–80% and approximately 6%, respectively, with some studies reporting pneumonitis as a negative prognostic factor for survival [18,57,59,60]. Pneumonitis is a particularly concerning immune-related adverse event in patients with stage III lung cancer, as prior radiotherapy may act synergistically to increase its risk of occurrence [61]. In addition, patients with lung cancer may already have compromised lung function and comorbid conditions. Together, this makes the differential diagnosis of immune-related pneumonitis difficult. To optimize patient outcomes, patient education on pneumonitis symptoms, and early diagnosis and intervention with an MDT, is essential [61]. Re-challenge with durvalumab can be considered after resolution of symptoms; however, guidelines on safely re-challenging are limited [60]. In two small retrospective studies, re-challenge with durvalumab was shown to be feasible, leading to pneumonitis recurrence in 14 and 29% of patients [59,60]. To optimize patient outcomes, the decision of durvalumab re-challenge should be made with an MDT.

## 3. Conclusions

There are many challenges related to logistics, communication, and education that can interfere with optimal delivery of care for patients with stage III unresectable NSCLC (Table 1); however, specific challenges will differ across jurisdictions. Therefore, to ensure efficient and patient-centered healthcare delivery, there is a need for regional- and institutional-level evaluation of care pathways across Canada, and globally. In the diagnosis and treatment planning stages of a patient’s cancer journey, access to timely assessments, an organized triage and referral system, and strong and consistent communication between HCPs, allied health professionals, and patients, are key to ensure eligible patients with stage III NSCLC can receive curative treatment options. Multidisciplinary team collaboration continues to be imperative not only in pre-treatment stages, but also during treatment management to optimize outcomes for these patients.

## Figures and Tables

**Table 1:** Challenges and strategies for optimizing clinical care for patients with stage III unresectable NSCLC.

Management Stage	Potential Challenges	Strategies for Optimization
Diagnosis and staging	Timely access to tests and results	Communication with referring physicians about expediting staging investigations that are required at time of referral. Use of triage nurse or centralized referral centres to ensure timely and organized patient workup investigations and timely referrals to appropriate specialists. Education of local referring physicians on how to refer patients to lung diagnostic assessment programs. Initiation of quality assurance investigations at individual centres to identify unique process inefficiencies
Treatment planning	Defining optimal treatment for patients borderline resectable/borderline eligible for cCRT	Implement regular MDT meetings to discuss challenging cases and/or establish strong communication lines between specialists for case discussions as needed.
Chemoradiation: initiation and management	Organizing delivery of cCRT for patient in rural/remote regions	Ensure oncologist and/or allied health professional awareness of patient support programs offering overnight lodging and transportation for patients when required.
	Interruptions to radiation treatment	Ensure treatment planning with contingency protocols based on common reasons for treatment delay. Follow recommendations to compensate for unscheduled treatment delays.
	Adverse events interfering with QoL and eligibility for durvalumab	Ensure close monitoring and swift management of adverse events during treatment.
Immunotherapy: initiation and management	Patients mentally/physically unprepared for durvalumab	Ensure discussion of durvalumab consolidation benefits at initial treatment planning stages (cCRT) to prepare patients.
	Requirement of CT for eligibility to consolidation durvalumab	Schedule CT scan ahead of time to align shortly after cCRT completion. Consider feasibility of alternatives to confirm non-progression such as chest x-ray or noting last cone beam findings.
	Uncertainty in role of different physicians during follow-up	Discuss responsibilities upfront between physicians involved in patient care.
	Managing pneumonitis	Patient education on pneumonitis symptoms, and early diagnosis and intervention guided by an MDT.
